# Supplementary material for: The Evolution and Characterization of the RNA Interference Pathways in Lophotrochozoa
Source: Genome Biol Evol. 2024 May 7;16(5):evae098. doi: 10.1093/gbe/evae098 (PMC11114477; doi:10.1093/gbe/evae098)
Supplement: evae098_Supplementary_Data [file evae098_supplementary_data.zip › Supplementary_materials.zip!/selected_miRNAs/NoGe_selected_miRNAs/mirdeep_pdf_files/NMRB01000231.1_Notospermus_geniculatus_strain_Ushimado_scaffold231,_whole_genome_shotgun_sequence_7807.pdf]

Provisional ID : NMRB01000231.1_Notospermus_geniculatus_strain_Ushimado_scaffold231,_whole_genome_shotgun_sequence_7807
Score total : 1243.9
Score for star read(s) : 3.9
Score for read counts : 1234.9
Score for mfe : 2.1
Score for randfold : 0
Score for cons. seed : 3
Total read count : 2434
Mature read count : 2425
Loop read count : 0
Star read count : 9

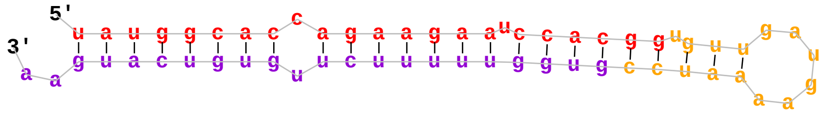

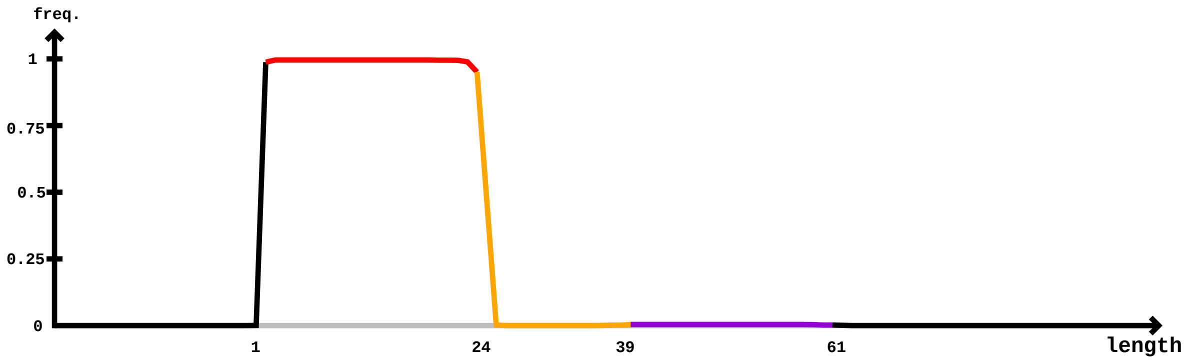

Mature Star

```
5'- uuugcuuuugucgugccauuuauggcaccagaagaauccacgguguugaugaaaauccgugguuuuucuugugucaugaauggucaugauuaggucgcuucgagugcuuugug  -3'   obs
    uuugcuuuugucgugccauuuauggcaccagaagaauccacgguguugaugaaaauccgugguuuuucuugugucaugaauggucaugauuaggucgcuucgagugcuuugug        exp
    ...((((..((((((((((((((((((.(((((((.((((((.(((......))))))))))))))))).))))))))))))).)))))).)))).((.......))......   reads   mm   sample
    ....................uauggcacUagaagaauccac........................................................................   1       1    Mm3
    ....................uauggcaccagaagaauccac........................................................................   2       0    Mm3
    ....................uauggcaccagaagaauccacg.......................................................................   8       0    Mm3
    ....................uauggcaccagaagaauccacgA......................................................................   1       1    Mm3
    ....................uauggcaccagaagaGuccacgg......................................................................   1       1    Mm3
    ....................uauggcaccagaagaauGcacgg......................................................................   2       1    Mm3
    ....................uauggcacAagaagaauccacgg......................................................................   1       1    Mm3
    ....................uauggcaccagaagaauccacgg......................................................................   181     0    Mm3
    ....................uauggcaccagaagaaCccacgg......................................................................   1       1    Mm3
    ....................uauggcaccagaagaauccacgU......................................................................   2       1    Mm3
    ....................uauggcaccagUagaauccacgg......................................................................   1       1    Mm3
    ....................uauggcaccagaagaauUcacgg......................................................................   1       1    Mm3
    ....................uauggcaccagaagaauccacggA.....................................................................   1       1    Mm3
    ....................uauggcaccagaagaauccacggu.....................................................................   10      0    Mm3
    .....................auggcaccagaagaauccacgg......................................................................   2       0    Mm3
    .....................auggcaccagaagaauccacggu.....................................................................   1       0    Mm3

    ....................uauggcaccagaagaauccac........................................................................   1       0    Fm3
    ....................uauggcacUagaagaauccac........................................................................   1       1    Fm3
    ....................uauggcaccagaagaauccacg.......................................................................   4       0    Fm3
    ....................uauggUaccagaagaauccacg.......................................................................   1       1    Fm3
    ....................uauggcacGagaagaauccacgg......................................................................   1       1    Fm3
    ....................uauggcaccagaagaauccacgg......................................................................   21      0    Fm3
    ....................uauggcacUagaagaauccacgg......................................................................   2       1    Fm3
    ....................uauggcaccagaagaaCccacgg......................................................................   1       1    Fm3

    ...................Cuauggcaccagaagaauccacgg......................................................................   1       1    Mm2
    ....................uauggcaccagaagaauc...........................................................................   2       0    Mm2
    ....................uauggcaccagaagaaucca.........................................................................   1       0    Mm2
    ....................uauggcacUagaagaauccac........................................................................   8       1    Mm2
    ....................uauggcaccagaagaauGcacg.......................................................................   1       1    Mm2
    ....................uaugUcaccagaagaauccacg.......................................................................   1       1    Mm2
    ....................uauggcaccagaagaauccacg.......................................................................   44      0    Mm2
```

```
                    Mature                                Star
uuugcuuuugucgugccauuuauggcaccagaagaauccacgguguugaugaaaauccgugguuuuucuugugucaugaauggucaugauuaggucgcuucgagugcuuugug
....................uUuggcaccagaagaauccacgg......................................................................   1      1   Mm2
....................uauggcaccagaagaauccacgg......................................................................   1482   0   Mm2
....................uauggcaccagaaAaauccacgg......................................................................   1      1   Mm2
....................uauggcaccagaagaGuccacgg......................................................................   1      1   Mm2
....................uauggcacUagaagaauccacgg......................................................................   2      1   Mm2
....................uauggcaccagaaUaauccacgg......................................................................   1      1   Mm2
....................uauggcaccGgaagaauccacgg......................................................................   2      1   Mm2
....................uauggcaccagaGgaauccacgg......................................................................   5      1   Mm2
....................uauggcaccagaagaaCccacgg......................................................................   3      1   Mm2
....................uauggcaccagaagaauUcacgg......................................................................   8      1   Mm2
....................uauggcaccagaagaaAccacgg......................................................................   4      1   Mm2
....................uauggcaccaAaagaauccacgg......................................................................   1      1   Mm2
....................uauggcaccagaagaaucUacgg......................................................................   1      1   Mm2
....................uauggcaccagaagaauccacgC......................................................................   3      1   Mm2
....................uauggcaccagaagaauAcacgg......................................................................   20     1   Mm2
....................uauggcaccagaagaauccacgU......................................................................   9      1   Mm2
....................uauggcaccagaagaauccacgA......................................................................   4      1   Mm2
....................uauggcaccagaagaauGcacgg......................................................................   6      1   Mm2
....................uauggcaccagaagaaGccacgg......................................................................   3      1   Mm2
....................uauggcaccagaagaUuccacgg......................................................................   1      1   Mm2
....................uauggcaccagaagaauccaUgg......................................................................   1      1   Mm2
....................Nauggcaccagaagaauccacgg......................................................................   1      1   Mm2
....................uauggcaccagaUgaauccacgg......................................................................   1      1   Mm2
....................uauggcaccagaagaauAcacggu.....................................................................   2      1   Mm2
....................uauggcaccagaagaauccacggA.....................................................................   1      1   Mm2
....................uauggcaccagaagaauccacggu.....................................................................   40     0   Mm2
....................uauggcaccGgaagaauccacggu.....................................................................   1      1   Mm2
....................uauggcaccagaagaauccacgguU....................................................................   4      1   Mm2
.....................auggcaccagaagaauccacg.......................................................................   1      0   Mm2
.....................auggcaccagaagaauccacgg......................................................................   6      0   Mm2
.....................auggcaccagaagaauccacggu.....................................................................   1      0   Mm2
.......................................................uccgugguuuuucuugugucau....................................   1      0   Mm2
..........................................................gugguuuuucuugugucaug...................................   1      0   Mm2
..........................................................gugguuuuucuugugucaugaa.................................   2      0   Mm2

....................uauggcaccagaagaauccacg.......................................................................   32     0   Mm1
....................uauggcaccaAaagaauccacgg......................................................................   1      1   Mm1
....................uUuggcaccagaagaauccacgg......................................................................   1      1   Mm1
....................uauggcaccagaagaauccacgA......................................................................   6      1   Mm1
....................uaugAcaccagaagaauccacgg......................................................................   1      1   Mm1
....................uauggcaccagaagaaAccacgg......................................................................   1      1   Mm1
....................uauggcaccagaagaauGcacgg......................................................................   1      1   Mm1
....................uauggcaccagaagaauccacgg......................................................................   212    0   Mm1
....................uauggcaccagaagaauAcacgg......................................................................   2      1   Mm1
....................uauggcaccagaagaaGccacgg......................................................................   1      1   Mm1
....................uauggcaccagaagaauccacggA.....................................................................   6      1   Mm1
....................uauggcaccagaagaauccacggu.....................................................................   9      0   Mm1
.....................auggcaccagaagaauccacgg......................................................................   8      0   Mm1
..........................................................gugguuuuucuugugucaugaa.................................   3      0   Mm1

....................uauggcaccagaagaauccacg.......................................................................   1      0   Fm1
....................Nauggcaccagaagaauccacgg......................................................................   1      1   Fm1
....................uauggcaccagaagaauccacgg......................................................................   118    0   Fm1
....................uauggcaccagaagaauUcacgg......................................................................   1      1   Fm1
....................uauggcaccagaagaauccacgU......................................................................   2      1   Fm1
....................uauggcaccagaagaauccacUg......................................................................   1      1   Fm1
....................uauggcaccagaagaauAcacgg......................................................................   1      1   Fm1
.....................auggcaccagaagaauccacgg......................................................................   1      0   Fm1
........................................................ccgugguuuuucuugugucaug...................................   3      0   Fm1

....................uauggcacUagaagaauccac........................................................................   1      1   Fm2
....................uauggcaccaCaagaauccacgg......................................................................   1      1   Fm2
....................uauggcaccagaagaaAccacgg......................................................................   1      1   Fm2
....................uauggcaccagaagaauGcacgg......................................................................   2      1   Fm2
....................uauggcaccagaagaauccacgg......................................................................   82     0   Fm2
....................uauggcaccagaagaauAcacgg......................................................................   1      1   Fm2
....................uauggcaccagaagaauccacggu.....................................................................   6      0   Fm2
....................uauggcaccagaagaauccacgguA....................................................................   1      1   Fm2
```
